# Supplementary material for: Tetrel Bonding and Other Non-Covalent Interactions Assisted Supramolecular Aggregation in a New Pb(II) Complex of an Isonicotinohydrazide
Source: Molecules. 2020 Sep 4;25(18):4056. doi: 10.3390/molecules25184056 (PMC7571010; doi:10.3390/molecules25184056)

checkCIF/PLATON report

You have not supplied any structure factors. As a result the full set of tests cannot be run.

THIS REPORT IS FOR GUIDANCE ONLY. IF USED AS PART OF A REVIEW PROCEDURE FOR PUBLICATION, IT SHOULD NOT REPLACE THE EXPERTISE OF AN EXPERIENCED CRYSTALLOGRAPHIC REFEREE.

No syntax errors found. CIF dictionary Interpreting this report

Datablock: shelx

```
Bond precision:  C-C = 0.0075 A          Wavelength=0.71073

Cell:           a=8.1660(11)    b=13.2850(17)     c=13.7842(18)
                alpha=90        beta=92.860(4)    gamma=90
Temperature:    179 K

                Calculated                  Reported
Volume          1493.5(3)                   1493.5(3)
Space group     P 21/n                      P 21/n
Hall group      -P 2yn                      -P 2yn
Moiety formula  C13 H11 N5 O3 Pb            C13 H11 N5 O3 Pb
Sum formula     C13 H11 N5 O3 Pb            C13 H11 N5 O3 Pb
Mr              492.47                      492.46
Dx,g cm-3       2.190                       2.190
Z               4                           4
Mu (mm-1)       11.316                      11.316
F000            920.0                       920.0
F000'           907.39
h,k,lmax        10,17,17                    10,17,17
Nref            3376                        3318
Tmin,Tmax       0.163,0.288                 0.220,0.346
Tmin'           0.085

Correction method= # Reported T Limits: Tmin=0.220 Tmax=0.346
AbsCorr = MULTI-SCAN

Data completeness= 0.983          Theta(max)= 27.321

R(reflections)= 0.0288( 2666)     wR2(reflections)= 0.0667( 3318)

S = 1.033                  Npar= 200
```

The following ALERTS were generated. Each ALERT has the format
test-name_ALERT_alert-type_alert-level.
Click on the hyperlinks for more details of the test.

Alert level G

```
PLAT004_ALERT_5_G Polymeric Structure Found with Maximum Dimension          1 Info
PLAT180_ALERT_4_G Check Cell Rounding: # of Values Ending with 0 =          3 Note
PLAT794_ALERT_5_G Tentative Bond Valency for Pb        (II)      .       2.13 Info
PLAT883_ALERT_1_G No Info/Value for _atom_sites_solution_primary .     Please Do !
```

```
   0 ALERT level A = Most likely a serious problem - resolve or explain
   0 ALERT level B = A potentially serious problem, consider carefully
   0 ALERT level C = Check. Ensure it is not caused by an omission or oversight
   4 ALERT level G = General information/check it is not something unexpected

   1 ALERT type 1 CIF construction/syntax error, inconsistent or missing data
   0 ALERT type 2 Indicator that the structure model may be wrong or deficient
   0 ALERT type 3 Indicator that the structure quality may be low
   1 ALERT type 4 Improvement, methodology, query or suggestion
   2 ALERT type 5 Informative message, check
```

It is advisable to attempt to resolve as many as possible of the alerts in all categories. Often the minor alerts point to easily fixed oversights, errors and omissions in your CIF or refinement strategy, so attention to these fine details can be worthwhile. In order to resolve some of the more serious problems it may be necessary to carry out additional measurements or structure refinements. However, the purpose of your study may justify the reported deviations and the more serious of these should normally be commented upon in the discussion or experimental section of a paper or in the "special_details" fields of the CIF. checkCIF was carefully designed to identify outliers and unusual parameters, but every test has its limitations and alerts that are not important in a particular case may appear. Conversely, the absence of alerts does not guarantee there are no aspects of the results needing attention. It is up to the individual to critically assess their own results and, if necessary, seek expert advice.

Publication of your CIF in IUCr journals

A basic structural check has been run on your CIF. These basic checks will be run on all CIFs submitted for publication in IUCr journals (*Acta Crystallographica*, *Journal of Applied Crystallography*, *Journal of Synchrotron Radiation*); however, if you intend to submit to *Acta Crystallographica Section C* or *E* or *IUCrData*, you should make sure that full publication checks are run on the final version of your CIF prior to submission.

Publication of your CIF in other journals

Please refer to the *Notes for Authors* of the relevant journal for any special instructions relating to CIF submission.

PLATON version of 26/06/2020; check.def file version of 17/06/2020

Datablock shelx - ellipsoid plot

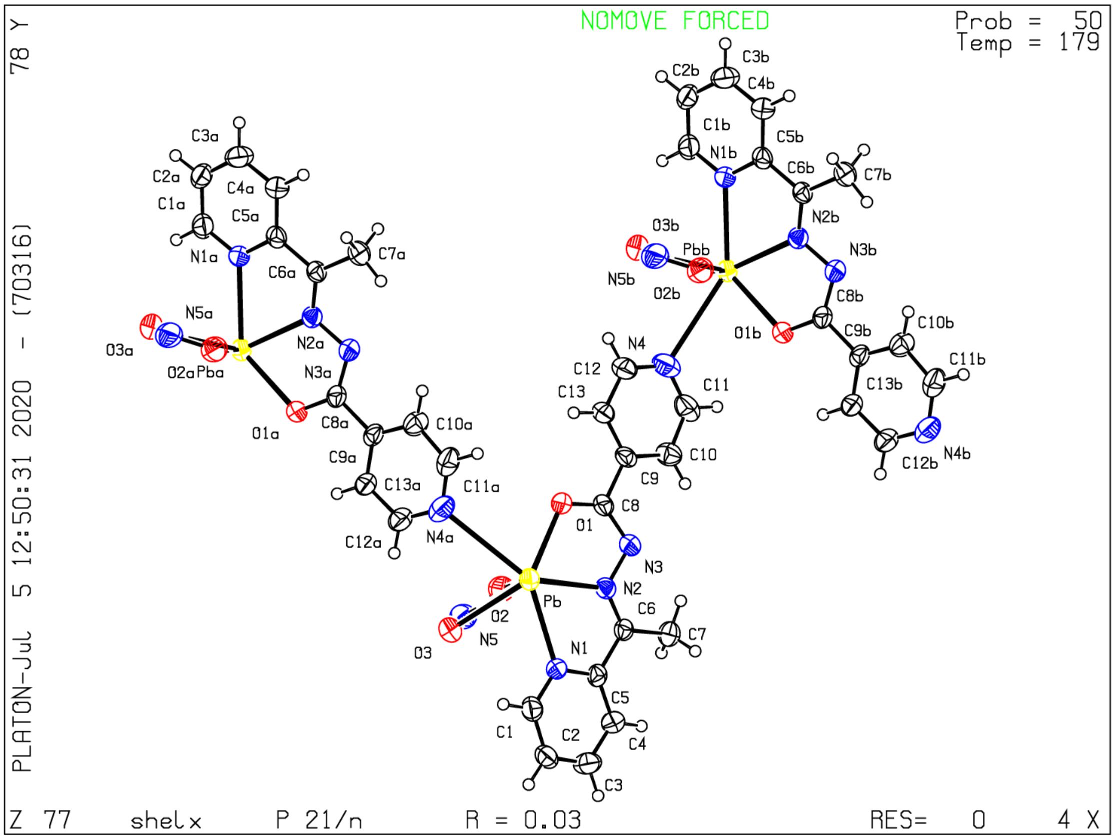

Supplement: Supplementary file 1 [file molecules-25-04056-s001.pdf]
